# Long-Range Low-Power Multi-Hop Wireless Sensor Network for Monitoring the Vibration Response of Long-Span Bridges †

**DOI:** 10.3390/s22103916

**Published:** 2022-05-22

**Authors:** Eleonora Maria Tronci, Sakie Nagabuko, Hiroyuki Hieda, Maria Qing Feng

**Affiliations:** 1 Department of Civil Engineering and Engineering Mechanics, Columbia University, New York, NY 10027, USA; mqf2101@columbia.edu; 2 Toshiba, Corporate Research & Development Center, Toshiba Corporation, Tokyo 105-0023, Japan; sakie1.nagakubo@toshiba.co.jp; 3 Toshiba America Inc., New York, NY 10020, USA; hiroyuki.hieda@toshiba.com

**Keywords:** long-range monitoring, low-power wireless, multi-hop network, accelerometers, long-span bridge, structural vibration monitoring, operational modal analysis, manhattan bridge

## Abstract

Recently, vibration-based monitoring technologies have become extremely popular, providing effective tools to assess the health condition and evaluate the structural integrity of civil structures and infrastructures in real-time. In this context, battery-operated wireless sensors allow us to stop using wired sensor networks, providing easy installation processes and low maintenance costs. Nevertheless, wireless transmission of high-rate data such as structural vibration consumes considerable power. Consequently, these wireless networks demand frequent battery replacement, which is problematic for large structures with poor accessibility, such as long-span bridges. This work proposes a low-power multi-hop wireless sensor network suitable for monitoring large-sized civil infrastructures to handle this problem. The proposed network employs low-power wireless devices that act in the sub-GHz band, permitting long-distance data transmission and communication surpassing 1 km. Data collection over vast areas is accomplished via multi-hop communication, in which the sensor data are acquired and re-transmitted by neighboring sensors. The communication and transmission times are synchronized, and time-division communication is executed, which depends on the wireless devices to sleep when the connection is not necessary to consume less power. An experimental field test is performed to evaluate the reliability and accuracy of the designed wireless sensor network to collect and capture the acceleration response of the long-span Manhattan Bridge. Thanks to the high-quality monitoring data collected with the developed low-power wireless sensor network, the natural frequencies and mode shapes were robustly recognized. The monitoring tests also showed the benefits of the presented wireless sensor system concerning the installation and measuring operations.

## 1. Introduction

The ability to robustly and reliably track the health conditions of structural systems over time has become a fundamental aspect of the development of resilient and safe urban communities and infrastructural systems. Consequently, in recent years, numerous methods have been developed for monitoring the structural health of bridges, buildings, and other civil engineering structures, many of which rely on the analysis of measured structural vibration response [1]. In particular, vibration-based monitoring strategies relying on parametric or non-parametric system identification techniques are among the most popular ones, as it emerges from the rich amount of publications in the research field [2,3]. The development of numerous semi-automated and automated approaches to carry on operational modal analysis has made handling and processing massive quantities of monitoring data easier [4,5,6,7,8]. This has automatically reflected in a lot of resources spent on developing robust and long-lasting sensor networks to acquire this tremendous amount of monitoring data that has been speeding up safely.

However, despite the significant research progress made in the past decade, the wide deployment of sensor-based Structural Health Monitoring (SHM) systems in civil engineering structures faces a major obstacle: the high cost of sensor installation and maintenance. However, battery-powered Wireless Sensor Systems (WSS) have a great potential to overcome this obstacle. Without requiring wiring for either power supply or signal transmission, such wireless sensors are far less expensive to install, particularly on large structures such as long-span bridges, compared to their wired counterparts. Moreover, the decrease in cost and dimensions of wireless sensors offers redundancy for important measurements and improves the accuracy of monitoring strategies.

These kinds of wireless networks result to be appealing for various and different applications in civil engineering. For example, WSS can easily be implemented to build sensor networks that meet the requirements of sudden event monitoring with minimal power consumption [9]. They can be exploited to handle system identification for densely instrumented infrastructures and structures [10]. They offer versatile networks that can be interfaced with a variety of sensors (accelerometers, strain gauges, thermistors, LIDAR, etc.) and used for bridge-weight in motion applications [11] or long-term monitoring of infrastructures [12].

In past years, the research community has spent important effort investigating and developing wireless sensor networks facing the major challenges associated with the use of WSS. In particular, wireless transmission of vibration data (usually at a high sampling rate) consumes a significant amount of power and requires frequent battery replacement. Moreover, the communication protocols adopted in the commercial platforms often do not cover wide monitoring areas. Therefore, a low-power, long-range wireless sensor network is needed for long-term, low-maintenance SHM of large-size civil engineering structures. The reader is referred to [13,14,15,16] for a complete state-of-the-art description of wireless technologies available in the literature.

Among the major WSS platforms that have emerged in recent years, Kane et al. [17] proposed the design of a new WSS platform, named Martlet, to address the demand for real-time application and high-speed onboard computation. However, the line-of-sight data transmission range covers less than 500 m, and the tests conducted using this technology all focus on short bridge systems or small areas. For example, Liu et al. tested the Martlet system [18] on an in-service pre-stressed concrete highway bridge covering a very small monitoring area, 64 m × 2.7 m. More recently, Lander et al. [11] implemented the Martlet wireless sensing system for performing bridge weigh-in-motion and structural health monitoring at an in-service short highway bridge. Moreover, the Martlet has relatively low-resolution data acquisition and inflexible power management schemes, which are inadequate for large-scale SHM applications.

AX-3D node developed by BeanAir, Inc. (Berlin, 12681, Germany) [19] is dedicated to vibration monitoring, especially in severe environments. The node features an innovative antenna diversity design to improve radio communication, remotely programmable filters, and supervision software (BeanScape) to provide real-time data visualization and automatic vibration analysis. This sensing system was tested for the acceleration response of the Tamar Bridge by Gaglione et al. [20] that experimented with a multi-hop vibration energy-harvesting wireless sensor network system built on top of the original proposal for bridge applications. However, even if they maintain energy-neutral operation, preserving energy with careful management of sleep and communication times, they only tested this network considering two sensors very close to each other (27 meters apart). Moreover, the AX-3D presents the downsides of having the same low resolution provided in the Martlet system and a milliseconds time synchronization error, resulting in false indications of structural damage in structural condition assessment.

The Waspmote v15, released in 2016 by Libelium, Inc. (Zaragoza, 50018, Spain) [21] has several attractive functionalities, such as over-the-air programming, multiple radio module choices, and cloud-based data management. The Waspmote v15 relies on the LoRa [22] communication protocol, one of the LPWA (Low Power Wide Area) sensor network technologies that realize low power consumption and long communication range. However, there is a limit to the size of data that can be sent at once, and the maximum data size is 250 bytes [23]. Therefore, it is not easy to collect acceleration waveform data by LoRa, which is about a hundred kbytes. Moreover, the Waspmote v15 was successfully used for environmental monitoring [24], but not yet for SHM applications for large-scale structures and infrastructural systems.

G-Link-200 from LORD Microstrain [25] is another commercial node well-suited for SHM. In particular, the sensor node supports 20-bit resolution data acquisition with extremely low noise and a wireless communication range of up to 2 km. The network was tested for monitoring the Old Liding$\ddot{o}$ Bridge that connects the island Liding$\ddot{o}$ to the city of Stockholm [26]. However, no information is provided in the system design or the monitoring application about the power consumption of this sensor system.

Zanelli et al. [27] proposed that the WindNode has been developed to be energetically autonomous through a balance between very low mean power consumption and the inflow of energy coming from the Photovoltaic panels. The achieved low energy consumption has been obtained by choosing suitable electronic components and implementing a state machine according to which the node stays in sleep mode most of the time. However, for wireless communication, they count on the Bluetooth Low Energy (BLE) protocol [28] which is a communication method that reduces the power consumption with respect to older communication options such as the original Bluetooth but allows to cover smaller monitoring areas. Their communication range is estimated to be less than 200 m.

In 2019, Fu et al. [29] assessed the efficacy of the Xnode developed by Spencer et al. in 2016 [30]. On the one hand, the results demonstrated that the Xnode is robust, capable of high-fidelity measurements, and efficient for long-term monitoring. On another, the power consumption requirements remain high. They proved that the communication range of the Xnodes extends from 200 m to over 1 km in the absence of obstacles or any particular obstruction. However, the ability of the network to keep this extent of communication range was only tested on a full-scale suspension bridge where the monitoring area covered by the single network was 250 m [30].

In response to the need to provide a network that offers to cover wide monitoring areas in a combination of low power consumption requirements, this work focuses on the development of a battery-powered wireless MEMS accelerometer system, which employs a low-power long-range multi-hop wireless networking technology referred to as the Low-Power Multi-Hop Network (LPMN) [31].

The proposed LPMN relies on a communication protocol that follows the IEEE802.15.4g/e, with a consequent sub-GHz wireless frequency. This allows longer communication lengths with respect to other commercial wireless sensor networks based on the BLE communication protocol that are proven to cover smaller areas [27]. Moreover, the LPMN grants a sufficiently long transmission data length, enabling the transfer of up to 900 bytes at once. Other common commercial networks rely on LoRa communication protocol [21] that can achieve longer communication distances but presents the strict limitation of a maximum of 222 bytes of data that can be sent at once. This feature makes the proposed LPMN more suitable for dealing with high-resolution data transmission, such as acceleration waveform data. In addition to the advantages mentioned, the proposed LPMN substantially reduces the power consumption requirements through a smart slot allocation for data transfer and an autonomously determined transmission path.

The proposed LPMN offers a flexible network that can be interfaced with different sensors and, therefore, collects various types of sensor data, such as the low-rate temperature and humidity data and high-rate acceleration data. Furthermore, the sensor nodes can be powered by batteries without requiring wired power supplies because of the low-power wireless. As a result, this wireless sensor system can be easily placed on large-size structures such as tall buildings or long-span bridges, getting read of costly cabling, and minimizing maintenance costs. To evaluate the robustness of the long-range, low-power wireless accelerometer system, a field test was performed on a long-span suspension bridge. Based on the measured bridge vibration data, an operational modal analysis of the bridge was carried out, and the identified bridge’s modal quantities were compared with those measured by conventional sensors in previous studies.

The design of the proposed sensor network is outlined in Section 2. Initially, in Section 2.1, an overall description of the network architecture is presented, followed by an illustration of the adopted transmission and data synchronization strategy. Then, in Section 2.2, the hardware components used to construct the network are described in combination with a short characterization of the data collection method. Finally, the proposed wireless sensor network validation results are shown in Section 3, where the network is used to carry on the vibration monitoring of the Manhattan Bridge. After briefly describing the Manhattan bridge, the experimental setup and installation process are presented, showing the advantages observed in the use of the proposed network. Next, the vibration monitoring of the acceleration response of the long-span bridge is described in Section 3.3, where the dynamic behavior of the structural system is characterized in terms of frequencies and modal shapes. To further validate the operational modal analysis results, they are finally compared with the state-of-the-art publications on the Manhattan Bridge.

## 2. Design of the Low-Power Wireless Sensor Network

This section describes the proposed low-power multi-hop network and the developed battery-powered wireless MEMS accelerometer system embedded into it.

### 2.1. Low-Power Wireless Multi-Hop Network

**Figure 1:**
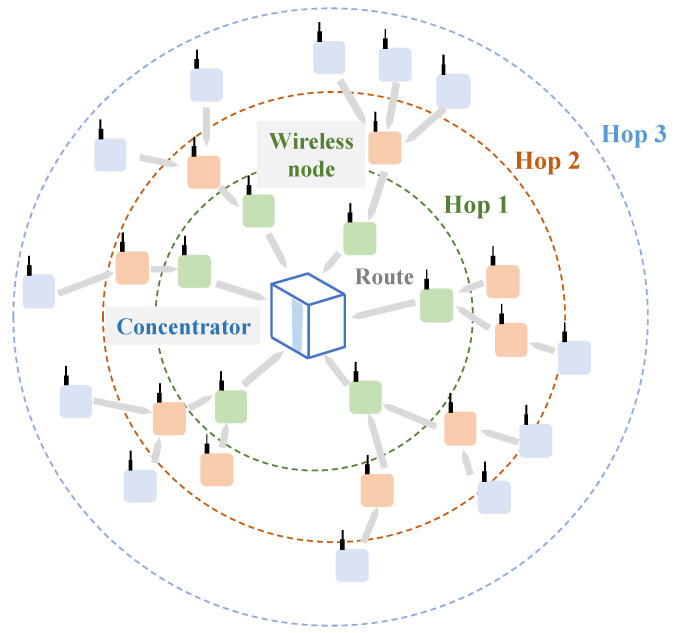
System organization of the Low-Power Wireless Sensor Network.

The LPMN (Figure 1) consists of multiple wireless nodes and a concentrator for gathering sensor data. The wireless nodes can be equipped with different sensor options giving the possibility to create and design a rich, diversified sensor network, customizable according to the specific application. Each sensor node communicates and transfers the collected sensor data straightly to the concentrator or a neighbor node. If the data are transmitted to another wireless node, that neighbor node forwards the acquired sensor data to another node or the concentrator. Therefore, by relaying the sensor data across *n* hops, these are finally gathered by the concentrator (Figure 1) as the final destination. A hop is the route a sensor data packet takes from one wireless sensor or intermediate point to another in the network. Consequently, for example, if the wireless node directly communicates with the concentrator, it computes a single-hop trip; if, instead, it needs to pass by an intermediate point, it engages a two-hop path to the concentrator, and so on. Furthermore, given that the wireless node adopts the sub-GHz frequency band to transmit and acquire data, with a communication protocol that follows the IEEE802.15.4g/e., the maximum straight communication distance or communication per hop of the LPMN in the line-of-sight environment is 1 km. Hence, the network can cover an extensive area by forwarding data between the wireless nodes.

The IPv6 Routing Protocol for Low-Power and Lossy Networks (RPL) [32] is one of the most common and representative communication methods used in wireless multi-hop networks [33]. In the multi-hop networks equipped with RPL, control information must be communicated in addition to sensor data to build and maintain a communication path within the network. However, the communication of both these information leads to an increase in the power consumption of the wireless nodes. In contrast, the low-power multi-hop network presents the benefit of having, for each transmission, the destination node of the network’s wireless node autonomously chosen. The determination of the route depends on the header part of the sensor data transferred by the wireless node and the Received Signal Strength Indicator (RSSI) when the data is acquired. Thus the wireless node can decide the destination node by self-sufficient distributed control without transferring control or path information, reducing the wireless node’s power consumption for creating and maintaining the communication route.

The proposed LPMN applies the Time Division Multiple Access (TDMA) procedure [34], which considers that the communication is executed at a specified time slot. Thus any possible interference or overlap between wireless nodes is averted by avoiding co-occurring communication timing between nodes. Consequently, the LPMN wireless node can send and receive at the assigned time and sleep at the unallocated time, efficiently saving power. Nevertheless, in a wireless multi-hop network that applies TDMA, the slot assignment strategy is strategic to achieve robust and efficient data transfer from the wireless node to the concentrator. The developed approach contemplates that, in the presence of data to be transmitted, the data is transferred in the slot that is usually in the sleep process to expand the data traffic. If there is no transmission data, the wireless node can sleep. Accordingly, the proposed LPMN employs an original slot allocation method to efficiently gather sensor measurement data and save power.

Commonly, in a wireless sensor network using TDMA technology [35], each time slot can be assigned to a node, and the transmission can be performed at that specific timing. However, this technology supports only the star topology network in which the node connects directly to the concentrator, and it cannot switch between transmission and sleep according to the data that must be transmitted. On the other hand, the proposed LPMN realizes not only a star network but also a multi-hop network, and the wireless node can switch between transmission and sleep according to the data that must be transmitted.

#### 2.1.1. Frame Structure and Slot Assignment

**Figure 2:**
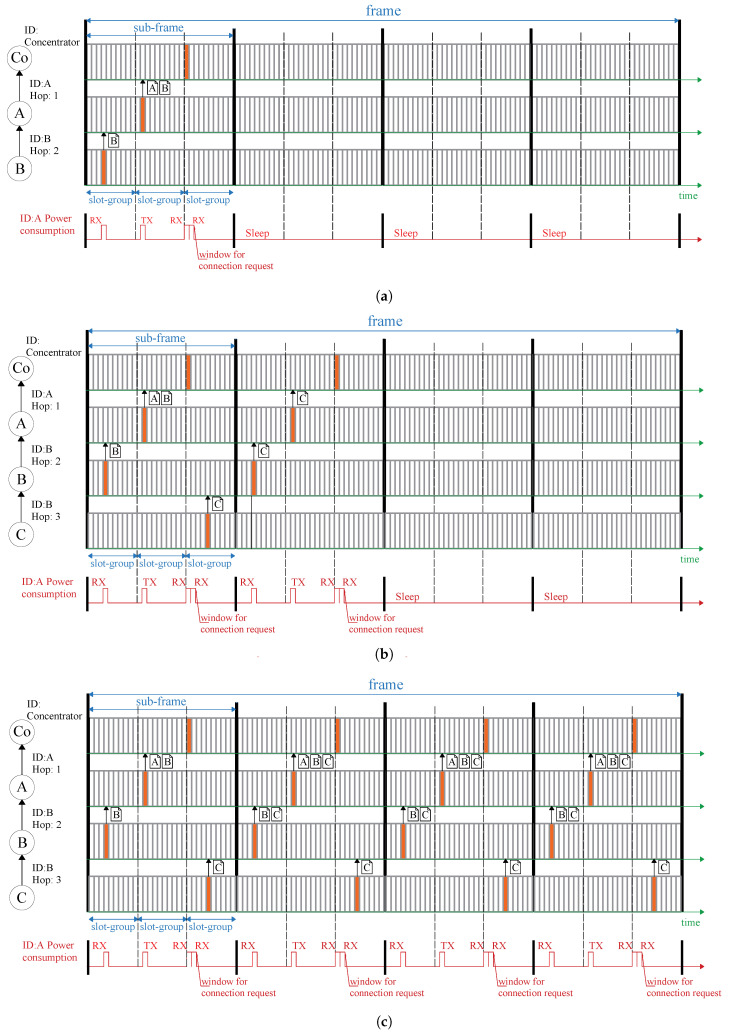
Frame configuration of LPMN: (**a**) when the maximum hop number is less than the *slot-group*number in a *sub-frame*; (**b**) when the maximum hop number is the*slot-group*number in a *sub-frame* or above; (**c**) when the wireless node has a large amount of sensor data.

Figure 2 displays the slot allocation strategy of the presented LPMN. Figure 2a illustrates that LPMN first defines a period named frame to manage the slot allocation approach. Then, one frame is divided into multiple *sub-frames*, where the single *sub-frame* is split into various *slot-groups*. Ultimately, the single *slot-group* is split into different slots. The wireless node’s data transmission and receiving times are allocated to the individual slot.

In a wireless multi-hop network, efficient data communication to the concentrator can be attained by transferring data, in sequence, from the wireless node farthest from the concentrator. In the LPMN, this stage is executed by distributing the transmission slot in accordance with the number of hops that separate the node from the concentrator and the ID of that wireless node. The number of hops and ID are always included in the header part of the transmission data, so no network pre-configuration is required. If the node is not connected to the network, the node receives data from neighboring nodes and selects a wireless node to communicate directly. Nodes connected to the network always send their hop numbers and IDs. Therefore, nodes can calculate their own hop numbers from the hop numbers they receive from the directly connected node. Following this approach, the node can autonomously determine the transmission timing.

For example, Figure 2a displays the slot allocation when the greatest number of hops in the network is two. In this case, the LPMN assigns the slot of the first *slot-group* of the first *sub-frames* to the node with two hops, then allocates the slot of the next *slot-group* to the right to the node located one hop away. Therefore, since wireless nodes with two hops can be transmitted, efficient data collection can be accomplished up to the terminal node (the concentrator). Furthermore, since the slots used by the wireless node in the *slot-group* are determined by the unique ID assigned to the node, the same slot will not be used unless the IDs are duplicated.

On the other hand, if the number of hops of the wireless node is equal to or greater than the number of *slot-group*s in the *sub-frame*, the *slot-group* to be used can be folded back. For example, if there is a wireless node with three hops, as shown in Figure 2b, the wireless node with three hops will use the rightmost slot in the *sub-frame*. If there is a wireless node with four hops, the wireless node with four hops will use the *slot-group* to the left of three hops. In this way, a node with one hop and a node with four hops share the same *slot-group*, but even if they belong to the same *slot-group*, they will not send in the same slot unless the IDs are duplicated. In addition, node A and node B must also relay the ID C data transmitted in the last *slot-group* of the first *sub-frame*. Therefore, node A and node B allocate the communication slot for transmission in the next *sub-frame*. By this method, the data of the wireless node having the number of hops equal to or more than the number of *slot-group*s in the *sub-frames* can also be relayed.

In such a manner, the LPMN can reroute data using as few transmissions as possible, even if a wireless node with many hops exists in the network. Nonetheless, the LPMN time slot size is 100 ms which imposes that the portion of data transferable within that period is restricted to 900 bytes. Accordingly, it is not feasible to transmit large-size data, such as waveform data, altogether. Therefore, the data must be separated and shared across numerous transfer slots. In the LPMN, the wireless node can keep sending data for each *sub-frame* until there is no data that need to be sent, as shown in Figure 2c. After that, the wireless node transmits only when measured sensor data needs to be sent. While, whenever there is no sensor data, it transfers only at the minimal needed timing shown in Figure 2a,b.

Whenever the LPMN is implemented in applications where the collected data consist of high-sampling-rate waveform data like acceleration measurements gathered from an accelerometer, the waveform data are transferred as soon as possible using multiple *sub-frames*. As a result, the wireless node saves power by sleeping in unnecessary transmit slots when the data transfer is concluded. Furthermore, when the LPMN is adopted in an application that involves gathering low-sampling-rate data with small data size, such as environmental measurements (temperature, humidity, etc.), the data sharing is terminated with only one transmission wireless node can sleep in the remaining time window. Hence, the LPMN slot allocation procedure can revise the transmission timing per the type and sampling requirements of the data to be collected, realizing efficient data collection and power-saving tailored to the application’s needs.

#### 2.1.2. Synchronization

It is possible to implement the TDMA method in an LPMN by synchronizing the timing of each wireless node. Furthermore, time synchronization between wireless nodes is required to compare the time waveforms of the sensor data from each node. Therefore, a power-saving time synchronization method of LPMN was developed, where the power consumption of the wireless node increases as the amount of transmission data increases.

The LPMN uses data that can be received from the destination node to perform time synchronization to achieve both power-saving and time synchronization. Each node adds time information to the header part of the sensor data, and the wireless node calculates the time difference from the destination node based on the time information of the data received from the destination node. The wireless node then corrects the internal clock with the time difference.

Synchronization messages are sent from the node that communicates directly to each wireless node. For example, a one-hop node that communicates directly with the concentrator receives synchronization data from the concentrator. The two-hop node receives synchronization data from the one-hop node that communicates directly. As the number of hops increases, the synchronization error also increases, but the synchronization error between one-hop nodes can be reduced to 135 μs by the power-saving time synchronization method of LPMN.

### 2.2. Battery-Powered Wireless MEMS Accelerometer System

**Figure 3:**
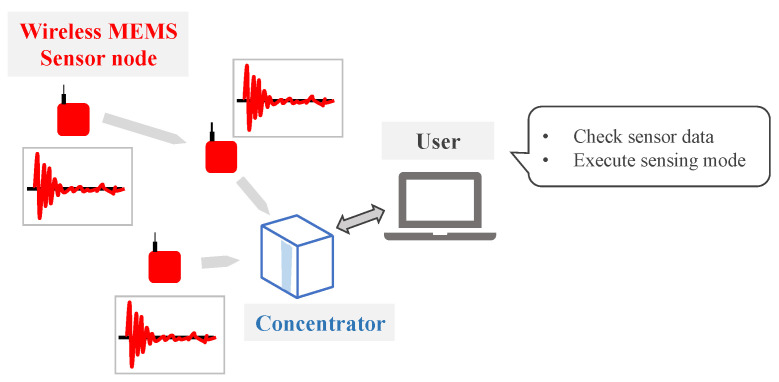
System structure of battery-powered wireless MEMS accelerometer system.

A battery-powered wireless MEMS accelerometer system is developed to monitor large-size structures comprising plural LPMN wireless sensor nodes and a concentrator (Figure 3). This section explains the adopted hardware in all its components and the system’s low-power acceleration collection method.

#### 2.2.1. Hardware Overview

**Figure 4:**
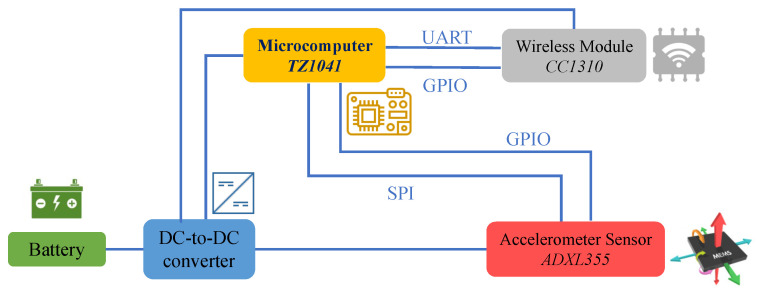
Functional scheme represents the main components present on the wireless sensor board.

**Figure 5:**
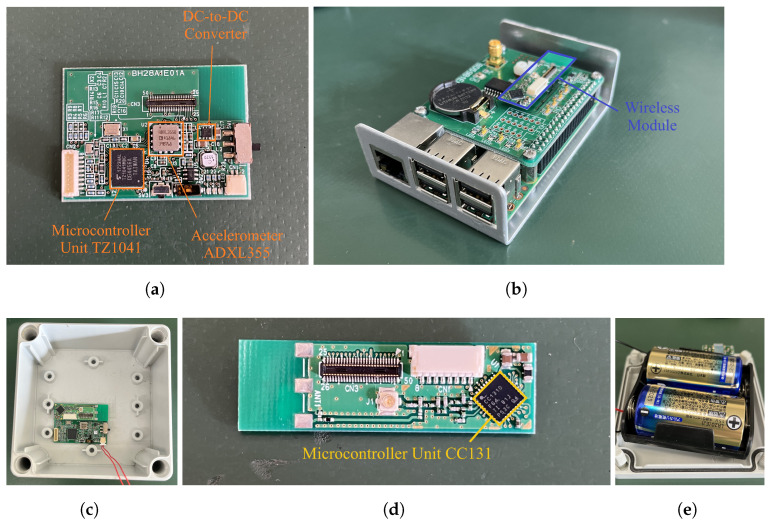
Hardware components: (**a**) wireless node main board; (**b**) concentrator; (**c**) view of the wireless node in the containing case, (**d**) the wireless module and the battery case (**e**).

Each wireless sensor node consists of a microcomputer that controls the wireless node, a wireless module that transmits and receives radio waves, an accelerometer, and a battery. Figure 4 shows the configuration of the wireless node. The microcomputer, wireless module, and the accelerometer of the wireless node can all be operated by the power supply of an ordinary cell battery (Figure 5e). Power is supplied via a DC-to-DC converter (Figure 5a). For the wireless module, the CC1310 microcontroller unit (Figure 5d) manufactured by Texas Instruments is adopted, which can communicate in the sub-GHz frequency band. In addition, the TZ1041 BlueTooth low energy transmitter manufactured by Toshiba was adopted as the microcomputer that controls the wireless node (Figure 5a). Finally, the ADXL355 manufactured by Analog Devices is employed for the MEMS accelerometer (Figure 5a).

The wireless node can use any accelerometer as long as it can send sensor data to the microcomputer via serial communication and has a function to notify if the acceleration measured by the sensor exceeds the threshold. Accelerometers with low current consumption are preferred. Various settings and sensor data acquisition are performed between the accelerometer and the microcomputer using the Serial Peripheral Interface (SPI) communication, which is a serial communication method, and the result of the accelerometer exceeding the threshold is notified to the microcomputer using the General-Purpose Input/Output (GPIO). The microcomputer can sleep and wake up when triggered by the GPIO connected to the accelerometer. Therefore, the wireless node can monitor vibrations with minimum power consumption.

The LPMN reduces power consumption by putting the microcomputer and wireless module into sleep mode. The accelerometer is turned on only when the measurement is needed. When the measurement is complete, the accelerometer is turned off or changed to the threshold-exceed notification mode. Since the wireless module cannot transmit or receive during sleep, it is necessary to synchronize the transmission timing between wireless nodes.

The concentrator (Figure 5b) that collects the acceleration data consists of a small Linux board equipped with the same wireless module as the wireless node. Since the concentrator always receives sensor data from wireless nodes, it cannot operate on batteries and requires a power source. When the concentrator gets the sensor data from the wireless node, it stores it in the internal storage of the Linux board. By connecting the concentrator to the cloud, it is possible to analyze the sensor data measured at a remote location using the cloud.

#### 2.2.2. Low-Power Acceleration Data Collection Method

Wireless sensor nodes equipped with accelerometers cannot operate on batteries for long periods due to increased power consumption when continuously measuring and collecting accelerometer data. Therefore, the wireless sensor node is designed to measure acceleration for a specific time period based on a trigger. There are two types of triggers: one is activated by the user, and another is turned on using the threshold-exceed notification mode of the accelerometer sensor.

Users can measure acceleration data whenever they want by activating a trigger. Once triggered, all wireless sensor nodes start measuring. On the other hand, the trigger that relies on the threshold-exceed notification mode starts the measurement when the acceleration exceeds the threshold.

Measuring starts with the trigger and stops when a fixed period elapses. Sending and receiving process continues until acceleration data is sent completely. After the acceleration data transmission is complete, the wireless sensor node only sends and receives wireless signals when necessary. Therefore, the wireless MEMS sensor node can realize low-power consumption. The concentrator saves the sensor data delivered by each sensor node. First, sensor data are stored in binary format. Then, once all the accelerometer data from the node are delivered, the binary data is transported to CSV data. Therefore, the user can check sensor data in the concentrator.

## 3. Field Experiment on a Long-Span Suspension Bridge

An ambient vibration test was conducted on the Manhattan Bridge in December 2020 to evaluate the performance of the wireless MEMS accelerometer network, including its measurement accuracy, reliability of the low-power multi-hop long-range wireless communications, and ease of installation. In addition, based on the measured acceleration data, the bridge structure’s vibration modal properties, including frequencies and mode shapes, were identified and validated by comparing with those obtained from previous studies.

### 3.1. Description of the Structure

The Manhattan Bridge is a landmark suspension bridge that crosses the East River in New York City, connecting Lower Manhattan at Canal Street with Downtown Brooklyn. Designed by Leon Moisseiff, the bridge began construction in 1901 and opened to traffic on 31 December 1909. The bridge presents an innovative design; indeed, it was the first suspension bridge to employ Josef Melan’s deflection theory for deck stiffening, resulting in the first use of a lightly webbed weight-saving Warren truss for its construction. Considered the forerunner of modern suspension bridges, it served as the model for many record-breaking bridges built in the first half of the twentieth century.

**Figure 6:**
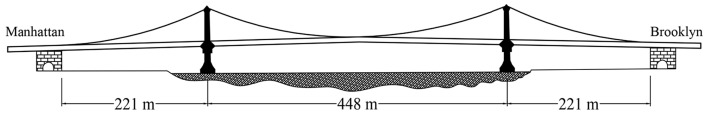
Picture and description of the section of the bridge.

**Figure 7:**
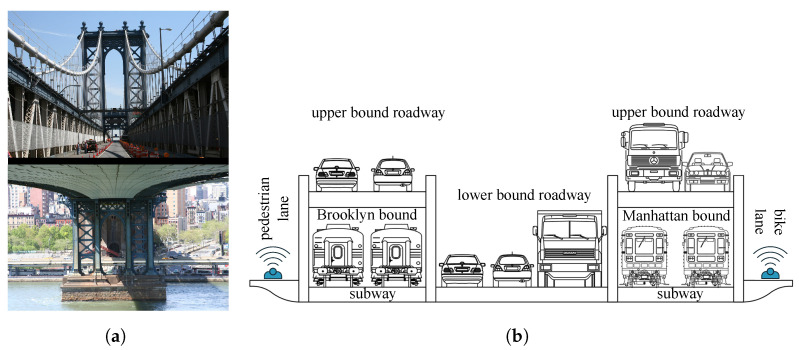
Picture (**a**) and description (**b**) of the section of the bridge.

The overall abutment to abutment length of the suspension bridge is 1767 m with two 221 m long side spans and a 448 m long main span (Figure 6). The width of the bridge is 37.3 m, while the overall depth of the two decks is approximately 7.3 m. It is supported by four main cables, 54.6 cm in diameter, connected through suspenders to Warren trusses with verticals. The two towers are 102 m high. Several members have built up riveted sections, especially those that remained original from the construction period as towers and trusses. These are composed of plates and angles riveted together. The bridge consists of a double-deck motorway (Figure 7) with four lanes on top and three lanes on the bottom that are designed to change direction when necessary to assist traffic flow. In addition to cars, the bridge carries four subway lines, a pedestrian lane, and a separate bikeway.

In 1982 the New York City Department of Transportation began an ambitious effort to rehabilitate the Manhattan Bridge, which included the reconstruction of the north and south upper roadways; rebuilding of the north and south subway tracks; installation of a truss stiffening system to reduce twisting effects on the deck; installation of a new north bikeway; and replacement of the lower roadway. Since 1982, the 109-year-old bridge, which crosses the East River connecting Lower Manhattan and Downtown Brooklyn, has been repaired 14 times, making this latest announcement the 15th construction contract.

### 3.2. Experimental Setup and Data Collection

#### 3.2.1. Sensor Locations and Wireless Networks

The ambient vibration test was conducted on the Manhattan Bridge in December 2020, in which ten wireless sensor units, each containing a triaxial MEMS accelerometer, were placed at the bridge deck to measure the bridge vibration under normal operational conditions involving passing subway trains and automotive vehicles as well as wind loads.

**Figure 8:**
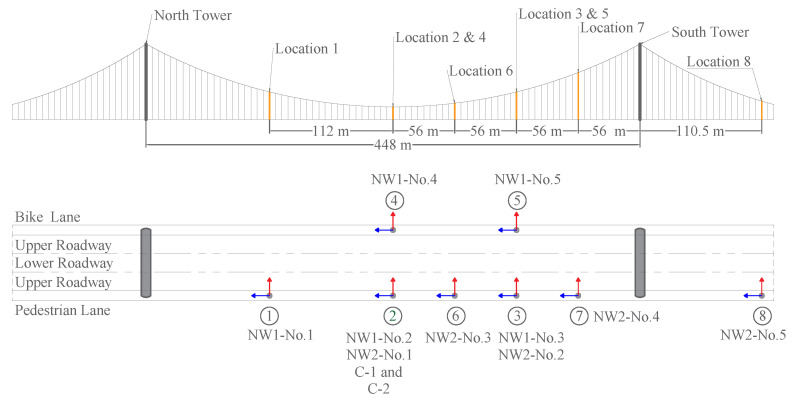
Sensors’ location along the bridge.

Figure 8 shows the eight locations of the ten wireless sensor units on the bridge deck. The sensor units were placed on the pedestrian lane (south side) and the bike lane (north side) of the bridge deck, as shown in Figure 7 and Figure 8. These sensor locations were determined based on a preliminary analysis to capture the bridge’s major vibrational mode shapes, including the torsional mode.

**Figure 9:**
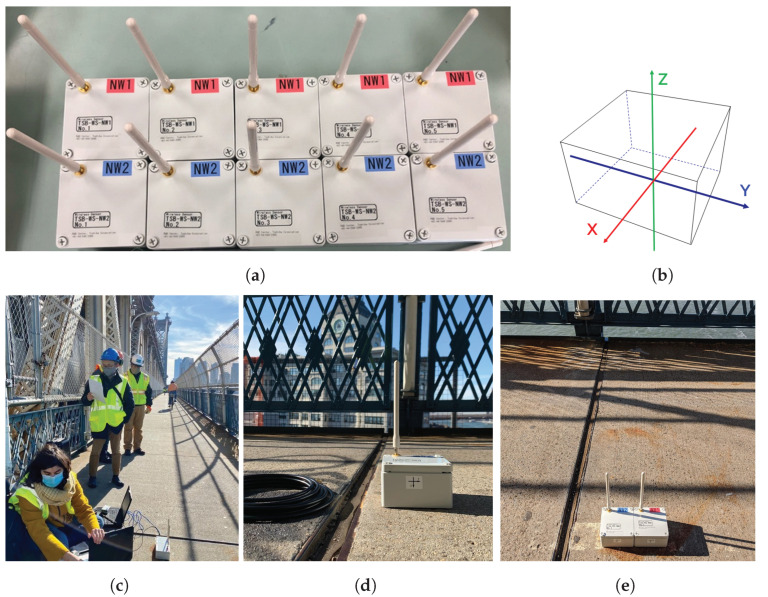
Sensor units for the field test: (**a**) the ten sensors in two wireless networks (NW1 and NW2); (**b**) description of the sensor axes; (**c**) sensor location ② during the field test; (**d**) a sensor unit during the test; (**e**) two sensors placed in the same location for synchronization.

The sensors were organized in two wireless networks labeled NW1 and NW2, as shown in Figure 8 and Figure 9. To increase the data sampling rate, the 10 wireless nodes were into 2 networks; each network contains five sensor units and reports back to a single concentrator, NW1 to concentrator C-1 and NW2 to concentrator C-2. Both concentrators were placed at location ② (Figure 8). Each concentrator is connected to a laptop computer. Two sensor units belonging to each network were placed in location ① and location ② to synchronize the two sensor networks. Figure 8e is a photo showing the sensor location ② during the test. The concentrators were also placed in this location.

Each sensor unit contains a triaxial MEMS accelerometer ADXL355 manufactured by Analog Devices, a wireless module and antenna, and two D batteries. The *y*-axis of the accelerometer is in the longitudinal direction of the bridge, parallel to the bike and pedestrian lane lines. The *x*-axis is in the bridge’s transverse direction, and the *z*-axis is in the vertical direction (Figure 9).

#### 3.2.2. Observation of Advantages of the Wireless Sensor System

The installation of the ten sensor units at the pre-determined locations and the setup and activation of the wireless networks took less than 45 min. The sensor units were taped to the ground using a thin layer of double-sided tape, which took less than a minute for each sensor unit. This field test demonstrated the ease of installation/setup of the wireless sensor system on a long-span bridge without interfering with its service, a major advantage over conventional sensors that require lengthy heavy cables and costly installation.

This field test also demonstrated the reliability of the networks’ wireless communications over the bridge’s steel truss structures. To measure the bridge’s torsional mode, sensor units were placed on both sides of the bridge (on the bike lane and pedestrian lane, respectively), as shown in Figure 7 and Figure 8. In sensor network 1 (NW1), two sensor units, NW1-No. 4, and NW1-No.5, were placed on the bike lane (the north side) of the bridge, across the steel truss structures from the other NW1 sensor units and concentrator C-1 that were placed on the pedestrian lane (the south side) of the bridge. The distance of concentrator C-1 was 37.7 m from sensor unit NW1-No.4 and 116.6 m from sensor unit NW1-No.5. Again, in between, there were the truss structures, as shown in Figure 7, and there was a concern that they might block wireless signals. Upon completion of the sensor installation and network setups, the network connectivity was tested to ensure that all the sensors were correctly reachable and visible by the concentrators for the activation, and sensor data were reliably transmitted to the concentrators. It was confirmed that all the sensor units in NW1 were reliably connected to concentrator C-1, despite the obstacle presented by the steel trusses composing the bridge’s deck. All the sensor units in the second network NW2, except for sensor NW2-No.5 at location ⑧, were reliably connected to concentrator C-2. Sensor NW2-No.5, placed on the side span, was 334 m away from concentrator C-2. The slope of the bridge side span caused the elevation of the sensor to be much lower than the concentrator. In other words, there was no line of sight between Sensor NW2-No.5 and concentrator C-2, which, combined with the 334 m distance, made it difficult to establish a reliable wireless connection. In addition, the steel truss South Tower (Figure 8) may have also blocked the wireless communication between this sensor and concentrator C-2. However, this issue was immediately solved by adding an extension cable for sensor unit NW2-No.5, allowing to place the external antenna in a higher location. Therefore, the different placing of the antenna allowed to efficiently overcome the transmission issues and collect reliable data from sensor unit NW2-No.5.

#### 3.2.3. Evaluation of Power Consumption

**Figure 10:**
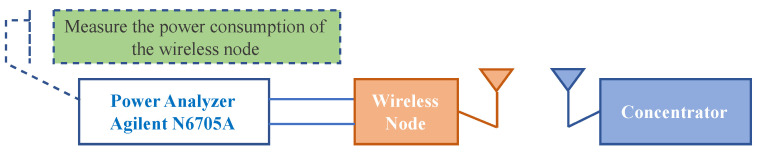
Power consumption test set-up.

A test was conducted to evaluate the power consumption of the wireless sensor node when communicating with the concentrator. A power analyzer was used, as shown in the test setup in Figure 10.

**Figure 11:**
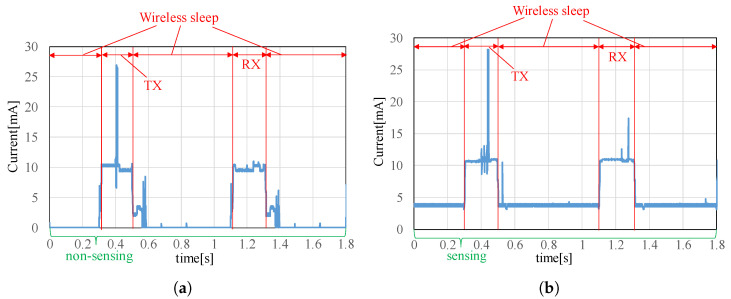
Power consumption result:(**a**) non-sensing, (**b**) sensing.

Figure 11a,b show the current time histories measured during sensing and non-sensing time periods, respectively. Table 1 tabulates the mean current consumption measured during transmitting, receiving, wireless sleeping with the sensor on (sensing), and wireless sleeping with the sensor off (non-sensing). Wireless sleep means that the microcomputer and the wireless module are in sleep mode. The test results show that the current consumption reduced to 29.28 μA from 3.73 mA during the wireless sleep period when the accelerometer was not sensing. Therefore, the developed wireless sensor node can operate on a battery for a long time by implementing the wireless sleep mode and turning off the sensor during non-sensing periods. Therefore, this wireless sensor system is useful for long-term monitoring with scheduled periodic measurements and temporary vibration tests like this Manhattan Bridge test.

### 3.3. Identification of the Bridge Dynamic Properties from the Vibration Measurements

For one day, six vibration records, 10 min long each, were collected at a sampling frequency of 31.25 Hz under the bridge’s normal operational conditions, including the cold winter weather and excitations caused by wind, passing trains, and automotive vehicles. In total, 30 channels of acceleration data were collected from the ten triaxial sensor units, representing the bridge vibration at the sensor locations in the longitudinal, transverse, and vertical directions.

The recorded acceleration response data of the suspension bridge were processed and analyzed. The resulting dynamic properties of the bridge, including natural frequencies and mode shapes, were compared with those obtained from previous field tests to evaluate the accuracy of the wireless sensor system developed in this study.

#### 3.3.1. Measured Bridge Vibration Data

**Figure 12:**
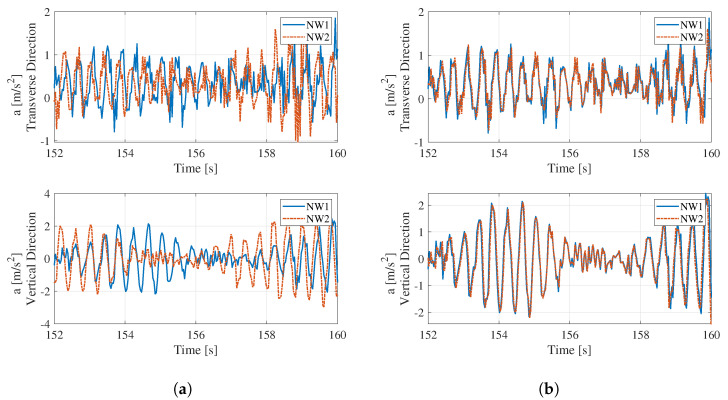
Acceleration data from two the sensors at location ②: (**a**) pre-synchronization, (**b**) post-synchronization.

The first step of the data processing was to synchronize the data measured from the two sets of wireless networks because proper identification of the bridge’s vibration mode shapes requires that the vibration data obtained from the sensors at various locations be synchronized. The sensor data are synchronized by matching the acceleration time histories measured by the two sensor units placed next to each other. Figure 12 illustrates the process. The two sensor units linked to the two different wireless networks NW1 and NW2, were placed next to each other at the sensor location ②. Figure 12a shows the acceleration time histories measured by the two sensors in the transverse and vertical directions. Because these two sensor networks were not triggered at the exact time, their time histories do not match each other. However, after adjusting the time to match the peaks in the acceleration time history data, the data from the two sensors match perfectly, as shown in Figure 12b.

#### 3.3.2. Identified Bridge Frequencies and Mode Shapes and Comparison with Previous Studies

The monitored bridge was excited by a combination of the passing subway train and automotive vehicle loads as well as wind loads. Unlike wind and vehicle loads commonly modeled as white noise excitation, moving train loads exhibit distinctive frequency characteristics related to their speed and the length of cars. There are four subway lines crossing over the Manhattan bridge: lines B, D, N, and Q. The N and Q trains on the south tracks are usually composed of ten 60-foot R160 cars, creating 11 loading cycles. The B and D trains on the north tracks have eight 75-foot-long R68 cars producing nine loading cycles. Multiple subway trains passed over the Manhattan bridge during the six 10 min long collected recordings. The combination of the train and vehicle loadings contributed to exciting the vertical modes of the bridge.

Analyzing in detail the recorded acceleration response of the bridge, the vibration data in the longitudinal direction exhibit an order of magnitude less power than the data in the other two directions. Therefore, modal analysis was conducted using the measured vibration data in the transverse and vertical directions. Moreover, being a long-span suspension bridge, the frequency content of the Manhattan bridge is concentrated in the lower frequency range and the operational modal analysis is carried on focusing on the frequency contents up to 1.0 Hz.

**Figure 13:**
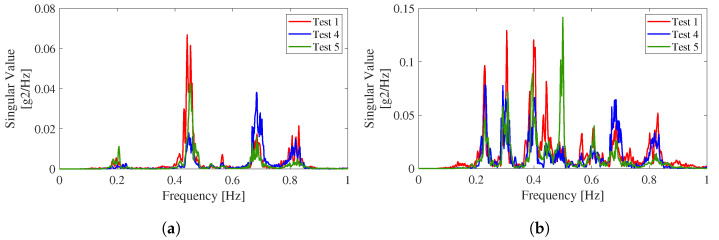
Singular values obtained from three tests: (**a**) transverse direction and (**b**) vertical direction.

The frequency-domain decomposition method [36] was initially applied to identify the bridge structure’s modal properties, including the natural frequencies and mode shapes. For example, Figure 13 shows the singular values obtained from the data measured during three of the tests. The vibration data in the vertical direction exhibit more peaks, i.e., more vibration modes, than those in the transverse direction.

**Figure 14:**
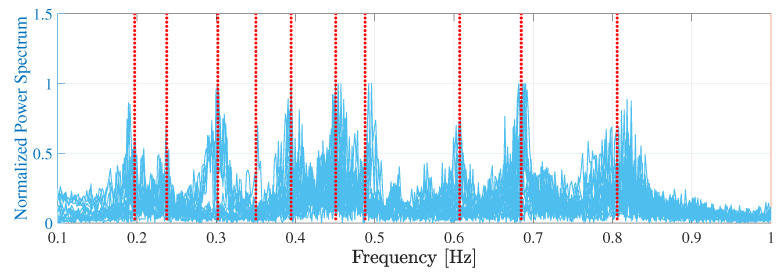
Normalized power spectrum and structural modes were identified using the semi-automated DD-SSI technique.

Then, to further validate the obtained results, the modal features were extracted from the system’s response using a semi-automated identification procedure based on Data-Driven Stochastic Subspace Identification (DD-SSI) technique developed by the authors. The reader is referred to [37] for a detailed description of the guidelines followed to pick the parameters’ ranges for the DD-SSI technique and to [7] for a step-by-step walk-through of the multi-stage semi-automated overall procedure. Figure 14 shows the structural modes identified adopting the DD-SSI technique with the normalized power spectrum in the background.

The adopted output-only technique consists of the following steps: (1) run the data analyses, using the classic DD-SSI formulation, for different values of (i) the order of the model, (ii) the number of output block rows, and (iii) the number of columns adopted to construct the Hankel matrix of the data, and (iv) the partition of the Hankel matrix in future and past outputs; (2) elimination of noise modes based on similarity checks between modal parameter estimates; (3) clustering of remaining modes; (4) outlier removal analysis, and (5) selection of the representative modal quantity. Figure 13 shows the singular values obtained from the data measured during three of the tests.

The natural frequencies and mode shapes corresponding to the first ten vibration modes of the bridge were robustly identified with the two adopted techniques. Table 2 tabulates the identified natural frequencies. This study identified eight vibration modes of the main span, including the three lateral modes (L), three vertical modes (V), and two torsional modes (T) of the bridge main span. In addition, two local modes for the bridge side span were identified as well: the first vertical mode (SV1) and the first torsional mode (ST1), which is coupled with the second torsional mode of the main span.

The identified bridge natural frequencies and mode shapes matched those extracted from previous field tests reported in the literature [38,39], validating the efficacy of the low-cost, low-power, long-range wireless MEMS accelerometer system. Furthermore, the high quality of the acceleration data measured by the low-power, low-cost, long-range wireless MEMS accelerometer system enabled the identification of a significant number of vibration modes with a very close frequency content.

**Figure 15:**
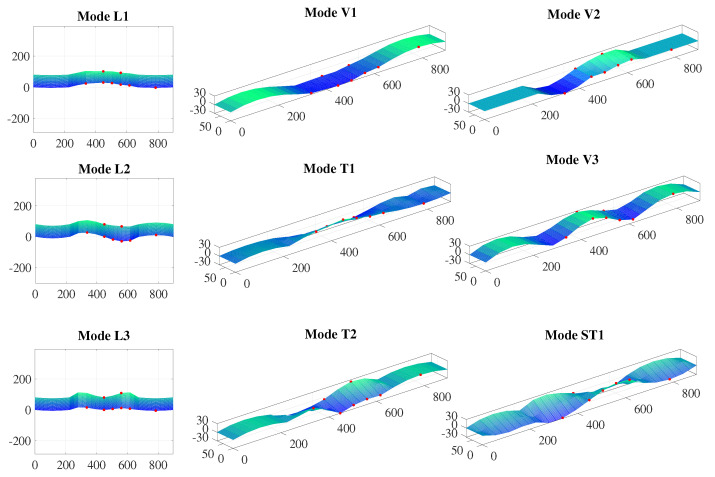
Identified modal shapes.

Figure 15 plots 3D representations of the identified vibration mode shapes corresponding to the frequencies, in which the red dots indicate the locations of the accelerometers. The magnitudes of the mode shapes at the sensor locations were identified from the measured data. The rest of the mode shapes were interpolated based on the mode shapes’ boundary conditions and the identified magnitudes at the sensor locations. First, the symmetrically located sensors along the longitudinal direction were used to examine whether the identified mode shapes were symmetric or not. Next, the sensors located at the same longitudinal location but at different transverse locations were used to determine whether the mode shapes of the bridge span were in phase or out of phase. Based on the symmetry and in-phase or out-of-phase motions of the mode shapes, the modal displacements at the sensor locations were projected to the other side of the bridge along with the longitudinal and transverse directions. Then, the projected modal displacements were measured modal displacements at the sensor locations, and the boundary conditions were used for the interpolation of the mode shapes.

As presented in Table 2, the identified bridge vibration modes from this study agree well with the results presented in previous studies [38,39]. The percentage in parenthesis in the table quantifies the differences between the frequencies identified in this analysis and the previous studies. Pantoli [38] adopted the bridge vibration data collected in an earlier field test by Mayer et al. [40] using interferometric radar and global positioning systems. The deflection time histories were collected simultaneously at 80 points along the midspan with a set-up and test time of about 3 to 4 h. Jang’s work is based on data measured by nine traditional wired sensors, Kinemetrics EpiSensor triaxial force balance accelerometers, where eight of those were placed at the same locations presented in this work.

The low-cost, low-power wireless MEMS accelerometer system was able to identify two more structural modes than the traditional, expensive wired force balance accelerometers presented by Jang et al. [39] and four more modes than the study [38]. Furthermore, the percentage discrepancies between the bridge frequencies obtained from this study and the two previous studies are below 5–6%. These differences could be caused by the fluctuations in the bridge frequencies given by operational and temperature seasonal variations. In summary, the bridge’s dynamic properties identified from the vibration data measured by the wireless MEMS accelerometer system matched the results from the previous tests using other types of sensors, which validated the efficacy of this battery-powered low-cost, long-range wireless sensor system for vibration tests of large-size civil engineering structures.

## 4. Conclusions

A wireless sensor system is highly desired for vibration monitoring of large-size structures due to the ease of sensor installation, but the long-range wireless transmission of high-sampling-rate vibration data consumes battery power. A low-power, long-range multi-hop wireless sensor system has been developed to address this challenge. The system is comprised of a concentrator and multiple battery-powered wireless nodes, each equipped with a low-cost 3-axis MEMS accelerometer. The sensor data from a sensor node are transmitted to the concentrate by hopping on neighbor nodes. Using sub-GHz radio communication, this low-power multi-hop network can achieve a maximum communication distance of 1 km in the line-of-sight environment. A number of efforts have been made to reduce the sensor nodes’ power consumption, including the implementation of wireless sleeping and turning off sensors while not measuring and eliminating the need for transmitting control signals to maintain wireless communications. Furthermore, this multi-hop wireless network has adopted an original slot allocation method that enables efficient sensor data collection while saving power.

The performance of the developed wireless sensor system was evaluated through a field vibration test on the landmark Manhattan Bridge in New York City. Ten battery-powered sensor nodes were placed at selected locations on the long-span suspension bridge deck to measure the vibration response of the bridge under normal operational loads, including traveling subway trains and automotive vehicles as well as wind loads. The field test demonstrated a number of remarkable advantages of the wireless sensor system for vibration measurement of a large size structure. It took less than 45 min to place the ten sensors on the long-span bridge deck. In addition, all the sensors except one (that was placed far away without line-of-sight due to the slope of the bridge deck) established reliable wireless communications over the bridge’s steel truss structures without any loss of sensor data.

The six vibration data sets, each 10 min long, collected from the field tests were analyzed, from which the dynamic properties of the bridge, including the natural frequencies and mode shapes of the first ten vibration modes, were successfully identified. They include the three lateral modes, three vertical modes, two torsional modes of the bridge main span, and one vertical and one torsional mode for the lateral span. The identified bridge natural frequencies and mode shapes matched those extracted from previous field tests reported in the literature, validating the efficacy of the low-cost, low-power, long-range wireless MEMS accelerometer system. Furthermore, the high quality of the acceleration data measured by the low-power, low-cost, long-range wireless MEMS accelerometer system enabled the identification of more vibration modes than those identified by the two previous vibration tests using a similar number of sensors. The wireless nature of the battery-powered MEMS sensor nodes allows an easy and flexible installation process. Combined with the long communication range and the low-power consumption, the developed wireless sensor system is ideal for monitoring large-size civil engineering structures and infrastructures.

## Figures and Tables

**Table 1:** Mean current and mean power consumption data results.

Time Period	Mean Current	Mean Power
Transmitting (TX)	10.13 mA	33.43 mW
Receiving (RX)	9.74 mA	32.14 mW
Wireless sleep (non-sensing)	29.28 μA	0.97 mW
Wireless sleep (sensing)	3.73 mA	12.31 mW

**Table 2:** Identified structural frequencies and comparison (percentage variation) with previous studies.

Frequencies								
Mode	Present Study		Pantoli			Jang		
	FDD	SA DD-SSI	et al. (2011)			et al. (2017)		
	[Hz]	[Hz]	[Hz]	FDD [%]	DD-SSI [%]	[Hz]	FDD [%]	DD-SSI [%]
L1	0.206	0.197	-	-	-	0.197	*4.37*	*0.00*
V1	0.223	0.237	0.233	*4.48*	*1.68*	0.227	*1.79*	*4.21*
V2	0.297	0.302	0.308	*3.70*	*1.99*	0.303	*2.02*	*0.33*
SV1	0.351	0.352	0.351	*0.00*	*0.28*	0.342	*2.56*	*2.84*
T1	0.381	0.394	0.391	*2.62*	*0.76*	0.373	*2.10*	*5.33*
L2	0.442	0.451	0.455	*2.94*	*0.89*	0.450	*1.81*	*0.22*
V3	0.488	0.489	-			0.490	*1.84*	*0.20*
ST1	0.602	0.607	-	-	-	-	-	-
T2	0.671	0.675	-	-	-	-	-	-
L3	0.824	0.806	-	-	-	-	-	-

## Data Availability

Not applicable.
